# Prognostic role of neutrophil-to-lymphocyte ratio in diffuse large B cell lymphoma patients: an updated dose–response meta-analysis

**DOI:** 10.1186/s12935-018-0609-9

**Published:** 2018-08-22

**Authors:** Shidai Mu, Lisha Ai, Fengjuan Fan, You Qin, Chunyan Sun, Yu Hu

**Affiliations:** 1 Institute of Hematology, Union Hospital, Tongji Medical College, Huazhong University of Science and Technology, Wuhan, 430022 China; 2 Cancer Center, Union Hospital, Tongji Medical College, Huazhong University of Science and Technology, Wuhan, 430022 China

**Keywords:** Neutrophil-to-lymphocyte ratio, Diffuse large B cell lymphoma, Prognosis, Meta-analysis

## Abstract

**Background:**

The neutrophil-to-lymphocyte ratio (NLR), a biomarker for systematic inflammation, has been recently identified as a prognostic factor for various types of both solid and hematologic malignancies. Here we conducted an updated dose–response meta-analysis to investigate whether NLR can be served as a prognostic biomarker in diffuse large B cell lymphoma (DLBCL).

**Methods:**

We systematically searched PubMed, Embase, ISI Web of Science and CNKI for relevant studies. Odds ratios or hazards ratios (HRs) with corresponding 95% confidence intervals (CIs) were pooled to estimate the association between NLR and clinicopathological parameters or survival of cancer patients.

**Results:**

Eleven trials with 2515 DLBCL patients were included in the meta-analysis. The results revealed that elevated pretreatment NLR was significantly associated with elder age, advanced Ann Arbor stage, higher incidence rate of B symptoms and bone marrow involvement, and higher lactate dehydrogenase level, etc. Moreover, increased NLR also predicted poorer overall survival (HR 1.826, 95% CI 1.238–2.692) and progression-free survival/event-free survival (PFS/EFS) (HR 1.591, 95% CI 1.124–2.252). And two-stage dose–response meta-analysis revealed non-linear association between increased NLR and risk of mortality in DLBCL patients.

**Conclusion:**

DLBCL patients with higher NLR are more likely to have poorer prognosis than those with lower NLR.

**Electronic supplementary material:**

The online version of this article (10.1186/s12935-018-0609-9) contains supplementary material, which is available to authorized users.

## Background

Diffuse large B cell lymphoma (DLBCL) is the most commonly occurring form of lymphoma, which occupies approximately 30–40% of preliminary diagnosed non-Hodgkin’s lymphomas (NHL). Despite the survival outcome of DLBCL patients has significantly improved [1] by the addition of rituximab immunotherapy to standard cyclophosphamide, doxorubicin, vincristine, and prednisone (CHOP) chemotherapy (R-CHOP) [2], approximately thirty percent patients with advanced stage of DLBCL remain intractable and the disease could relapse [3]. Prognostic assessment is important for designing of individualized therapy in DLBCL on the basis of accurate estimation of outcome.

A number of prognostic factors [4] have been studied, such as gene expression profiling [5], immunohistochemistry-based detection of prognostic biomarkers [6] and early interim analysis with positron emission tomography [7] following the initiation of R-CHOP therapy. However, many of these prognostic means are costly, difficult to perform, or not easily interpreted. Therefore, another prognostic models for DLBCL, which are inexpensive, widely available, and easily interpreted, are urgently needed for clinicians.

Recently, numerous studies have demonstrated that the ratio of different kinds of peripheral blood cells can be used to predict prognosis of lymphoma [8, 9], such as lymphocyte-to-monocyte ratio (LMR), neutrophil-to-lymphocyte ratio (NLR) and platelet-to-lymphocyte ratio (PLR), etc. Moreover, previous studies [10–13] have showed the relationship between increased pretreatment NLR and poor prognosis in different tumors. However, due to the divergence in the study design and sample size, direct impact of NLR level on DLBCL patients’ survival and tumor’s clinicopathological parameters remains inconclusive. In this study, we searched PubMed and Embase and Web of Science databases for the relevant studies and performed a meta-analysis in order to determine the association between NLR and some clinicopathological parameters, as well as the prognostic role of NLR in DLBCL.

## Materials and methods

### Search strategy and selection of studies

This meta-analysis was carried out in accordance with the standard guidelines for meta-analyses and systematic reviews of tumor marker prognostic studies [14, 15]. Relevant studies published before September, 2017 (date last searched), were identified through electronic searches using PubMed, Embase, and Web of Science databases. The following search terms were used: (1) “DLBCL” (e.g. “diffuse large B cell lymphoma” “B cell lymphoma” “lymphoma”); (2) “NLR” (e.g. “neutrophil to lymphocyte ratio” “neutrophil lymphocyte ratio” “neutrophil-to-lymphocyte ratio”); (3) “prognosis” (e.g. “outcome” “survival” “mortality” “recurrence”). Electronic searches were supplemented by scanning reference lists of articles identified for all relevant studies.

Studies were considered eligible if they met the following criteria: (1) patients were histopathologically diagnosed with DLBCL according to World Health Organization criteria [16]; (2) correlation of pretreatment NLR with overall survival (OS) or progression free survival (PFS) was reported; (3) Studies failing to report hazard ratio (HR) and 95% confidence interval (CI) were included with sufficient data for estimation [17]; (4) exclusion of abstracts, letters, reviews, case reports, and multiple published reports. When there were several reports concerning the same cohort we included the high quality and most recent publication in our meta-analysis.

All initially identified studies were screened of titles and/or abstracts; then full texts were retrieved for studies that satisfied all selection criteria. Each study was independently assessed for inclusion by two reviewers (Mu and Ai) and discrepancies within the reviewing pair were resolved by the third party (Sun and Hu).

### Data collection and extraction

We used a predesigned data abstraction form to extract relevant information. The following details were extracted: (1) the family name of first author, year of publication, country (region) of the population studied, sample size, patient age, gender, follow-up period; (2) clinicopathological parameters including Ann Arbor stage, etc.; (3) survival data including OS and PFS/EFS; (4) cut-off value for defining “elevated NLR”. OS was defined as the interval between the medical treatment and the death of patient or the last follow-up. PFS was calculated from the date of treatment until the detection of the recurrence tumor or death from any cause.

### Quality assessment

A quality assessment was independently performed in each of the included studies by two reviewers (Fan and Qin) using the Newcastle–Ottawa Quality Assessment Scale (NOS) [18]. This scale uses a star system (with a maximum of nine stars) to evaluate a study in three domains: selection of participants, comparability of study groups, and the ascertainment of outcomes of interest. We judged studies that received a score of seven or more stars to be at low risk of bias, and those that scored less than seven to be at high risk of bias. This cut-off point was chosen according to the distribution of relative quality scores of all included studies. Any disagreement was resolved by the third party (Sun and Hu).

### Statistical analysis

HR and 95% CI were obtained directly from each literature or from estimation according to the methods by Parmer et al. [17]. The combined rate (odds ratio, OR) and its 95% CI were used to evaluate the strength of association between NLR and clinicopathological parameters.

Heterogeneity among included studies was checked by the χ^2^-based Q test and I^2^ test. If there was no significant heterogeneity between studies (*p *> 0.10, I^2^ < 40%), the fixed-effect model was used. Otherwise, the random-effects model was chosen. All statistical tests were two sided and the significance level was set at 5%.

A two-stage dose–response meta-analysis was conducted to assess whether NLR was associated with higher risks of mortality from DLBCL, based on specific cut-off values, distribution of death cases and person years, and adjusted HRs with 95% CIs. We use the generalized least square regression described by Orsini et al. [19] to calculate the specific-study linear trend and 95% CIs for higher NLR within each study from the natural logs of adjusted HRs and 95% CIs, and pooled HRs and 95% CIs were obtained under the random-effects model. We approximately derived person years from follow-up duration and the number of participants at each NLR level. The midpoint of the higher NLR category was set at 1.2 times the lower boundary (specific cut-off value in each study). And we set the lower boundary to zero in the lower NLR category. We evaluated a potential curve linear association between NLR and risks of mortality from DLBCL, using restricted cubic splines with three knots at percentiles 10, 50, and 90% of the distribution [20].

Because characteristics of populations, ascertainment of cut-off value and adjustments for confounding factors were not consistent between studies, we further conducted a sensitivity analysis by removing one or several studies to explore possible explanations for heterogeneity and to examine the influence of various exclusion criteria on the overall risk estimate.

Assessment of publication bias was first evaluated by the Begg’s funnel plot, and then performed for each of the pooled study groups using Egger’s bias indicator test [21]. All analyses were carried out using STATA statistical software package version 14.0 (STATA, College Station, TX).

## Results

### Selection and characteristics of included studies

**Fig. 1:**
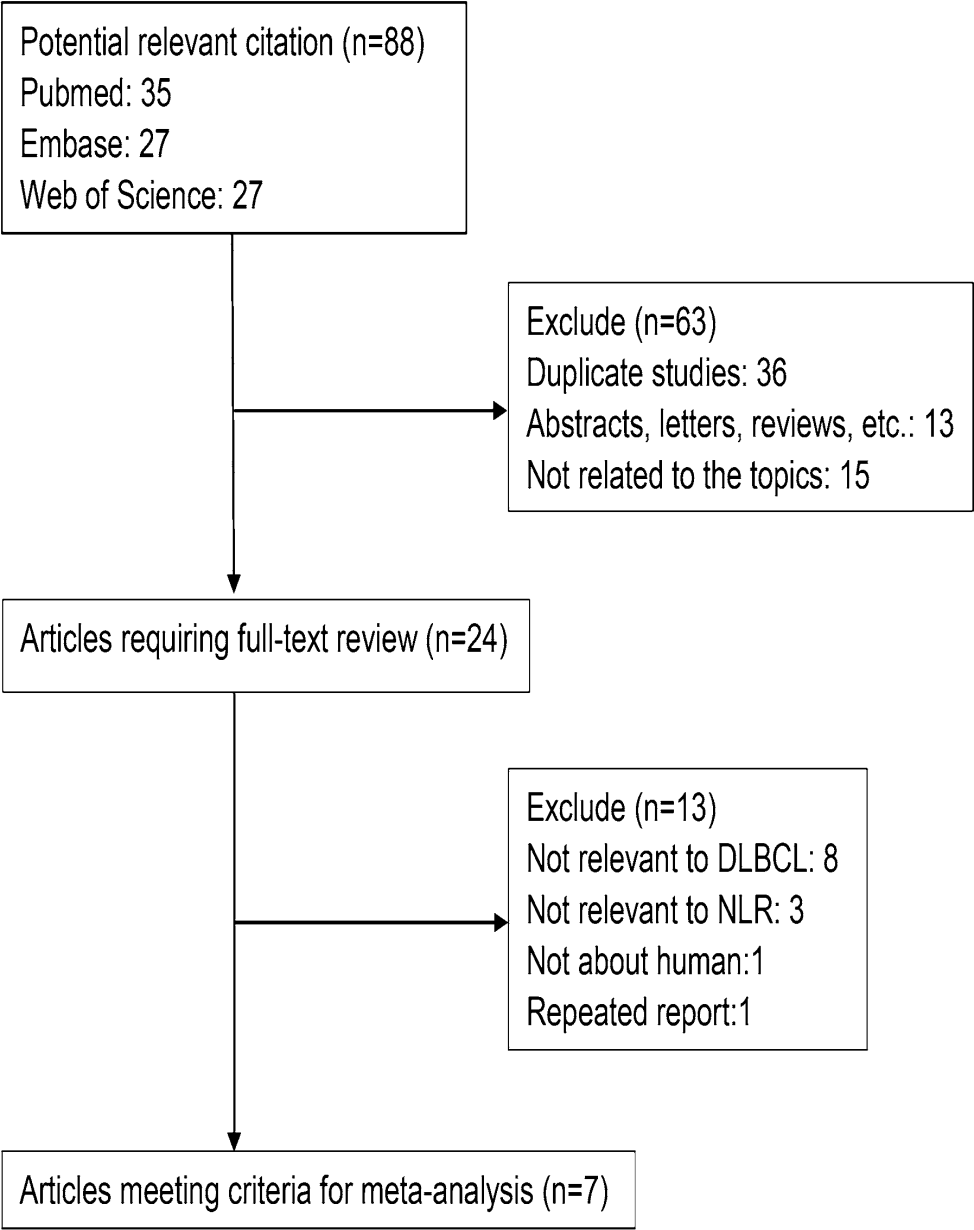
Flow diagram of selecting relevant published works regarding NLR in DLBCL

The flow diagram of the literature search was shown in Fig. 1. The initial search algorithm retrieved a total of 88 studies. After excluding the duplicates (n = 36); abstracts, letters, reviews, etc. (n = 13); and the studies not related to research topics (n = 15), the remaining studies (n = 24) were reviewed by reading the full text. Additional studies were then excluded because they were not relevant to DLBCL (n = 8), not relevant to NLR (n = 3), not clinical research about human (n = 1), or repeated report about the same cohort (n = 1). Therefore, 11 studies [8, 9, 11, 22–29] between 2010 and 2017 with a total of 2515 DLBCL patients were enrolled in our meta-analysis.

The characteristics of the included studies were summarized in Table 1. The publication periods of all included studies ranges from 2010 to 2016. Eight studies were from the eastern region (5 from China and 3 from Korea), and three from the western region (1 from the United States, 1 from Austria, and 1 from Croatia). Six studies enrolled < 200 patients and five studies had > 200 patients. 6 studies were at low risk of bias (NOS ≥ 7), the rest five studies were at high risk of bias (NOS < 7).

**Table 1:** Characteristics of studies included in the meta-analysis

Author	Year	Region	Follow-up (month)	Age (year)	No. (M/F)	Sample size	Outcome	Cut-off value	NOS score
Porrata et al. [28]	2010	USA	59.1 (13.9–107.8)	64 (20–92)	146/109	255	OS, PFS	3.5	8
Ho et al. [11]	2015	China	53.28	61.0 (16–88)	80/68	148	OS, PFS	2.11	7
Keam et al. [23]	2015	Korea	59.0 (2.5–118.5)	61 (16–87)	226/221	447	OS, PFS	3	8
Melchardt et al. [9]	2015	Austria	53	65.3 (20–92)	270/245	515	OS	5.54	8
Ming et al. [29]	2015	China	NR	55 (20–85)	24/27	51	OS	2.32	6
Hong et al. [22]	2016	Korea	48.9 (46.5–51.3)	56 (16–86)	177/136	313	PFS	2.42	8
Ni et al. [27]	2016	China	NR	54 (14–75)	36/23	59	OS, PFS	2.915	6
Periša et al. [25]	2016	Croatia	27 (1–105)	63 (22–87)	51/66	117	OS, EFS	2.63	6
Wang et al. [26]	2016	China	29 (2–122)	NR	90/66	156	OS, PFS	3.0	6
Park et al. [24]	2016	Korea	NR	60 (32–81)	53/46	99	OS, PFS	3.5	6
Wang et al. [8]	2017	China	NR	54 (18–86)	202/153	355	OS, PFS	2.81	7

*NR* not reported, *OS* overall survival, *PFS* progression free survival, *EFS* event free survival, *NOS* Newcastle–Ottawa Quality Assessment Scale

### Association between NLR and clinicopathological parameters

Among eleven studies in our meta-analysis, eight [8, 23–29] presented a significant association between NLR and Ann Arbor staging of DLBCL patients (AA III-IV vs. AA I-II: pooled OR 2.121, 95% CI 1.367–3.293), with significant heterogeneity (χ^2^ = 30.24, *p *< 0.001; I^2^ = 76.9%). Apparently, the study from Porrata et al. [28] might be the major source of the heterogeneity. After removing it from the enrolled studies, the pooled OR 2.477 (95% CI 2.013–3.049) without any heterogeneity (χ^2^ = 4.09, *p *= 0.664; I^2^ = 0%) indicated that higher NLR is significantly related to advanced Ann Arbor staging of DLBCL patients.

Moreover, we analyzed the association between NLR and other clinicopathological parameters, showing that higher NLR was significantly relevant to elder age, higher incidence rate of B symptoms and bone marrow involvement, poorer Eastern Cooperative Oncology Group-performance status (ECOG-PS), and higher lactate dehydrogenase (LDH) level (Table 2). However, the meta-analytic results presented us with no significant association between NLR and gender, extra nodal sites, and international prognostic index (IPI) score.

**Table 2:** Meta-analysis of the association between elevated NLR and clinicopathological parameters

Association	No. of studies	No. of patients	Pooled HR (95% CI)	Heterogeneity	
				I^2^ (%)	*p*-value
Ann Arbor stage	8	1525	2.121 (1.367–3.293)	76.9	< 0.001
IPI	7	1170	1.869 (0.881–3.962)	88.8	< 0.001
B symptoms	7	1474	1.867 (1.034–3.372)	84.3	< 0.001
BM involvement	4	1004	1.550 (1.148–2.094)	8.0	0.353
ECOG-PS	8	1525	2.786 (2.067–3.755)	0	0.673
Extranodal sites	7	1422	1.314 (0.809–2.133)	76.2	< 0.001
LDH	8	1525	2.911 (1.550–5.467)	88.7	< 0.001

*IPI* international prognostic index, *BM* bone marrow, *ECOG-PS* Cooperative Oncology Group performance status, *LDH* lactate dehydrogenase

### Association between NLR and survival

**Fig. 2:**
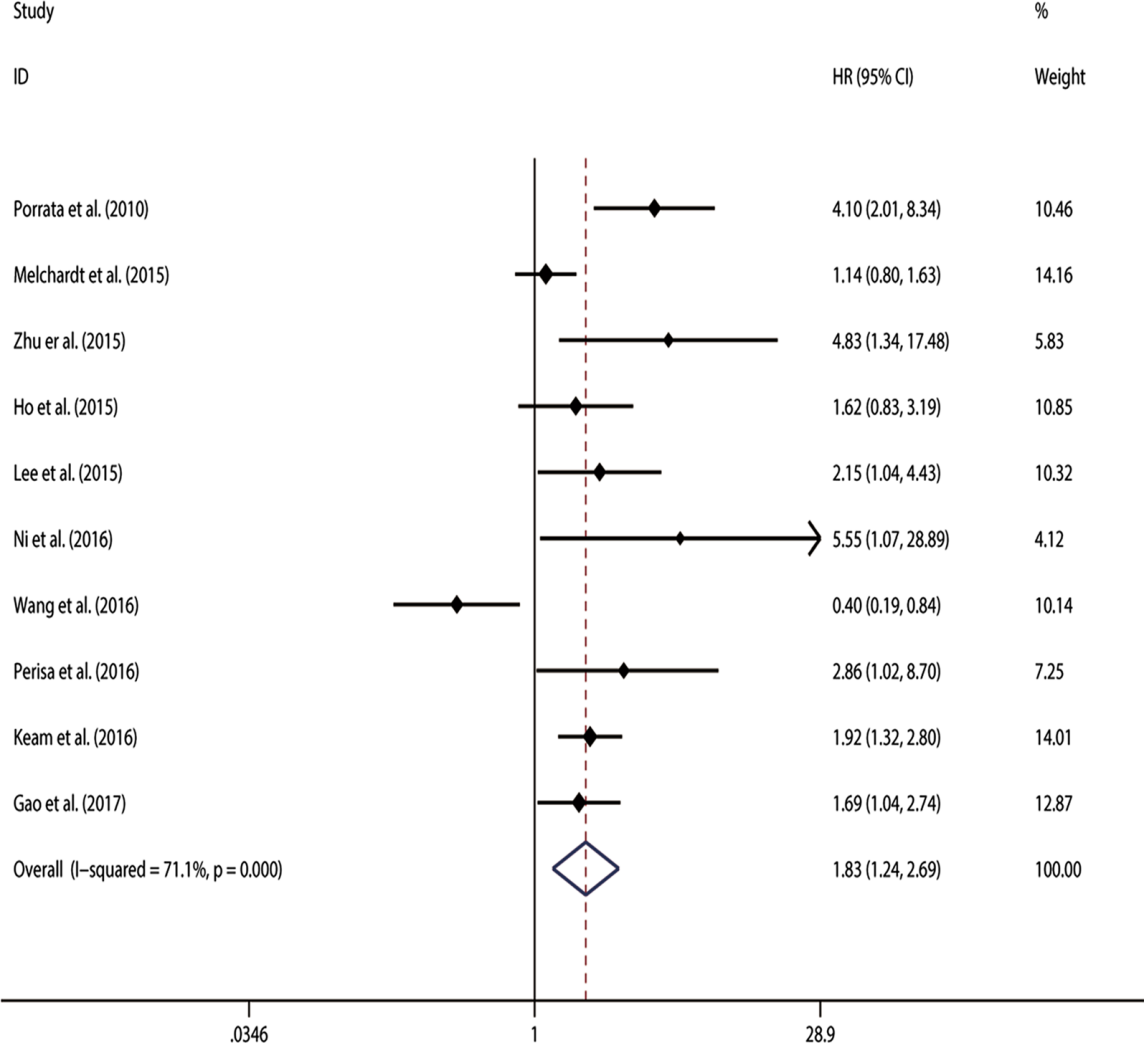
Meta-analysis of the association between elevated NLR and OS in DLBCL

11 studies [8, 11, 22–28] with 2454 patients in our analysis were included to examine the association between NLR and survival of DLBCL patients. With significant heterogeneity (χ^2^ = 19.74, *p *= 0.032; I^2^ = 49.3%), the pooled HR of 1.752 (95%CI 1.464–2.096, Fig. 2) indicated that patients with elevated NLR were expected to have shorter OS. Using restricted cubic spline function, we found non-linear associations between higher NLR and shorter OS in DLBCL patients (P_*non*-*linearity*_ = 0.007; ${\text{chi}}_{model}^{2}$ = 26.88).

**Fig. 3:**
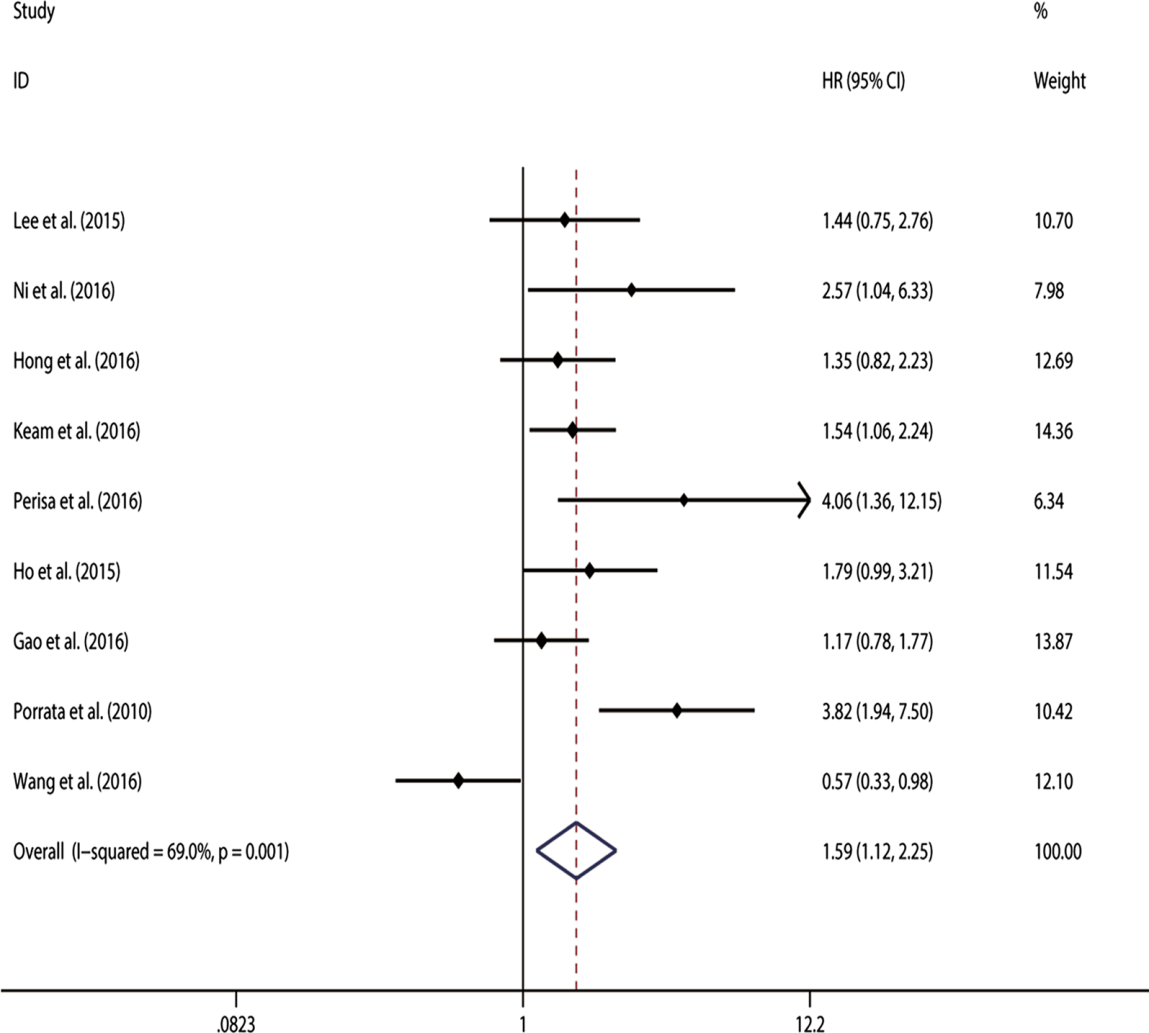
Meta-analysis of the association between elevated NLR and PFS in DLBCL

Furthermore, 9 studies [8, 11, 22–25, 27, 28] were pooled to estimate the association between NLR and PFS/EFS in DLBCL patients. The analytic results showed a significant correlation between high NLR and shorter PFS/EFS. (HR 2.495, 95% CI 2.208–2.819) without significant heterogeneity (χ^2^ = 15.36, *p *= 0.053; I^2^ = 47.9%) (Fig. 3). the dose–response meta-analysis showed a linear association between higher NLR and shorter OS in DLBCL patients treated with R-CHOP (P_*non*-*linearity*_ = 0.0011; ${\text{chi}}_{model}^{2}$ = 24.12).

As we all know, OS and PFS/EFS are correlated with each other, and split-and-merge results will increase the probability of type I error. Therefore, the 3-level random-effects meta-analysis model is applied using R software 3.2.2 [30] and meta SEM packages [31], verifying the overall effects of NLR on OS and PFS/EFS in DLBCL patients (HR 1.729, 95% CI 1.221–2.448).

In addition, we combined HRs through cox multivariate analysis, to explore the independent prognostic role of NLR for OS and PFS/EFS in DLBCL patients although there was significant heterogeneity across the studies, pooled HRs and corresponding 95% CIs of OS and PFS/EFS were 1.752 (1.464-2.096) and 1.739 (1.255-2.411), respectively.

To explore the source of heterogeneity, subgroup analysis and meta regression were performed by the study location (eastern vs. western region), sample size (≥ 200 vs. < 200), cut-off value defining “elevated NLR” (≥ 3 vs. < 3), and NOS score (≥ 7 vs. < 7). The subgroup analysis did not alter the prognostic role of NLR in OS substantially (Table 3), with significant heterogeneity across studies in most subgroups. However, subgroup analysis of PFS/EFS by the study location showed obvious decreased heterogeneity. And meta-regression analysis revealed that the study location (*p* 0.02) might explain the source of the heterogeneity across the studies on PFS/EFS (Table 4).

**Table 3:** Subgroup analysis and Meta regression of pooled HRs for OS in DLBCL patients with high NLR

Subgroup analysis	No. of studies	No. of patients	Pooled HR (95% CI)		Meta regression (*p*-value)	Heterogeneity	
			Fixed	Random		I^2^ (%)	*p*-value
Region							
Western	3	840	1.563 [1.149–2.126]	2.246 [0.888–5.681]		82.2	0.004
Eastern	8	1315	1.665 [1.317–2.105]	1.701 [1.050–2.756]	0.657	69.8	0.003
Sample size							
< 200	6	616	1.542 [1.077–2.209]	1.921 [0.894–4.127]	0.952	75.1	0.001
≥ 200	4	1539	1.659 [1.334–2.064]	1.841 [1.187–2.853]		72.6	0.012
Cut-off value							
< 3.0	5	823	2.539 [1.784–3.612]	2.908 [1.755–4.819]	0.060	36.8	0.176
≥ 3.0	5	1332	1.368 [1.098–1.705]	1.287 [0.795–2.084]		75.5	0.003
NOS score							
≥ 7	5	1687	1.656 [1.345-2.038]	1.787[1.242-2.571]		63.5	0.027
< 7	5	468	1.627 [1.350-1.960]	1.826[1.238-2.692]	0.949	80.1	0.000

*NOS* Newcastle–Ottawa Quality Assessment Scale

**Table 4:** Subgroup analysis and Meta regression of pooled HRs for PFS in DLBCL patients with high NLR

Subgroup analysis	No. of studies	No. of patients	Pooled HR (95% CI)		Meta regression (*p* -value)	Heterogeneity	
			Fixed	Random		I^2^ (%)	*p*-value
Region							
Western	2	358	3.885 [2.185–6.907]	3.885 [2.185–6.907]	0.027	0	0.926
Eastern	7	1577	1.299 [1.071–1.576]	1.313 [0.973–1.772]		54.8	0.039
Sample size							
< 200	5	565	1.320 [0.972–1.792]	1.588 [0.832–3.032]	0.850	75.6	0.003
≥ 200	4	1370	1.530 [1.209–1.742]	1.641 [1.092-2.465]		66.1	0.031
Cut-off value							
< 3.0	4	830	1.445 [1.209–1.742]	1.660 [1.048–2.629]	0.697	50.2	0.110
≥ 3.0	5	1105	1.449 [1.144–1.834]	1.511 [0.874–2.612]		79.8	0.001
NOS score							
≥ 7	5	1518	1.561 [1.262–1.931]	1.648 [1.183–2.297]		56.0	0.059
< 7	4	417	1.179 [0.824–1.688]	1.576 [0.674–3.688]	0.757	80.0	0.002

*NOS* Newcastle–Ottawa Quality Assessment Scale

### Sensitivity analyses

Sensitivity analyses were performed next. A single study involved in the meta-analysis was deleted each time to unveil the influence of the individual data set to the pooled HRs. In the current study, removing any of the included studies had no significant impact on the meta-analytic results, indicating the robustness of the results (Additional file 1: Figure S1).

### Publication bias

The Begg’s funnel plot showed no significant asymmetry for all included studies (Additional file 2: Figure S2). Accordingly, the *p* value of Egger’s test indicated that there was no publication bias among the studies included in our analysis (*p *= 0.792 for OS, and *p *= 0.792 for PFS/EFS).

## Discussion

Recently, several studies have shed light on the association between NLR and prognosis of cancer patients. However, these results are not comparable because of the heterogeneity on design and population, and the diversity in cut-off values defining “elevated NLR”. A previous meta-analysis combining 9 studies has shown that NLR was a significant indicator for poor OS and PFS from a total of 2297 individuals [32]. Our study is an updated meta-analysis covering a total of 11 published studies with 2515 patients reporting the independent prognostic role of NLR in DLBCL patients treated with R-CHOP. Moreover, we conducted a dose–response meta-analysis to assess the association between NLR and risk of mortality from DLBCL among different cut-off values in the included studies. Because OS and PFS/EFS are correlated with each other, the 3-level random-effect meta-analysis model is applied to assess the prognostic role of NLR in DLBCL patients.

Several recent studies have demonstrated that the inflammatory nature of a tumor’s microenvironment is a critical component of tumor initiation, growth, and progression [33, 34]. Furthermore, a variety of systemic inflammatory biomarkers have been identified and studied in patients with DLBCL, such as NLR [35], serum LDH [36], serum C-reactive protein (CRP) [37], serum albumin [38], etc. NLR has the advantage of low economic cost and wide availability, thereby drawing increasing attention. Mechanically, an elevated NLR is usually caused by neutrophilia and lymphopenia. Neutrophilia may lead to secretion of vascular endothelial growth factor (VEGF) and suppression of cytolytic activity of immune cells such as lymphocytes, natural killer cells, and activated T cells, thus accelerating tumor progression [39, 40]. Lymphopenia indicates disease severity and is linked to the immune escape of tumor cells from tumor-infiltrating lymphocytes (TILs) [41, 42]. Therefore, an elevated NLR generates a favorable immune microenvironment, thereby correlating to poor prognosis of patients.

The meta-analysis had some limitations that called for cautious interpretation of the results. First, only 11 studies published in full-text were included in this meta-analysis. Some information was missed and individual patient data were unattainable, which might decrease the accuracy of the results. Second, the cut-off value defining high NLR varied among individual studies (Table 1), which may have contributed to heterogeneity. Third, differences of paper quality and sample size across the studies might cause bias in the meta-analysis although subgroup analysis and meta regression did not show the paper quality or sample size as the resource of heterogeneity. Forth, most of the included studies reported positive results, therefore our results might overestimate the prognostic significance of NLR to some degree. However, there was no significant publication bias of the enrolled studies on OS or PFS/EFS.

Despite the above limitations, our meta-analysis supports the values of NLR as a promising independent risk factor of survival in DLBCL patients. NLR can be easily obtained from routine blood tests, thus intermediate assessments about changes in NLR during therapy were simply available. Keam et al. revealed that a reduction in NLR after R-CHOP therapy in patients with high pre-NLR was associated with better prognosis. Therefore, combining NLR with the IPI score helps to identify higher risk patients who need additional consolidation therapy, such as autologous stem cell transplantation. That is, NLR can help personalize the treatment intensity, as well as aftercare schedule, in order to increase the likelihood of early detection.

## Conclusion

Here, we searched electronic databases for relevant studies, and enrolled 11 studies with a total of 2515 patients for meta-analysis, drawing a conclusion that patients with higher NLR were more likely to have poorer prognosis than those with lower NLR. Taken together, the results from our meta-analysis suggest that NLR gains a prognostic value for patients with DLBCL. More multi-center prospective cohorts are warranted to further validate the role of the NLR in DLBCL.

## Additional files

**Additional file 1: Figure S1.** Funnel plot of publication bias for (A) OS and (B) PFS.

**Additional file 2: Figure S2.** Sensitivity analysis of studies concerning NLR and (A) OS and (B) PFS.

## Abbreviations

NLR: neutrophil-lymphocyte ratio
DLBCL: diffuse large B-cell lymphoma
OR: odds ratio
HR: hazards ratio
CI: confidence interval
LDH: lactate dehydrogenase
OS: overall survival
PFS: progression-free survival
EFS: event-free survival
NHL: non-Hodgkin’s lymphomas
CHOP: standard cyclophosphamide, doxorubicin, vincristine, and prednisone chemotherapy
R-CHOP: rituximab immunotherapy to standard CHOP chemotherapy
ECOG-PS: Eastern Cooperative Oncology Group-performance status
NOS: the Newcastle–Ottawa Quality Assessment Scale
VEGF: vascular endothelial growth factor
TIL: tumor-infiltrating lymphocyte
IPI: international prognostic index
