# Supplementary figures and images for: Prognostic role of neutrophil-to-lymphocyte ratio in diffuse large B cell lymphoma patients: an updated dose–response meta-analysis
Source: Cancer Cell Int. 2018 Aug 22;18:119. doi: 10.1186/s12935-018-0609-9 (PMC6103859; doi:10.1186/s12935-018-0609-9)

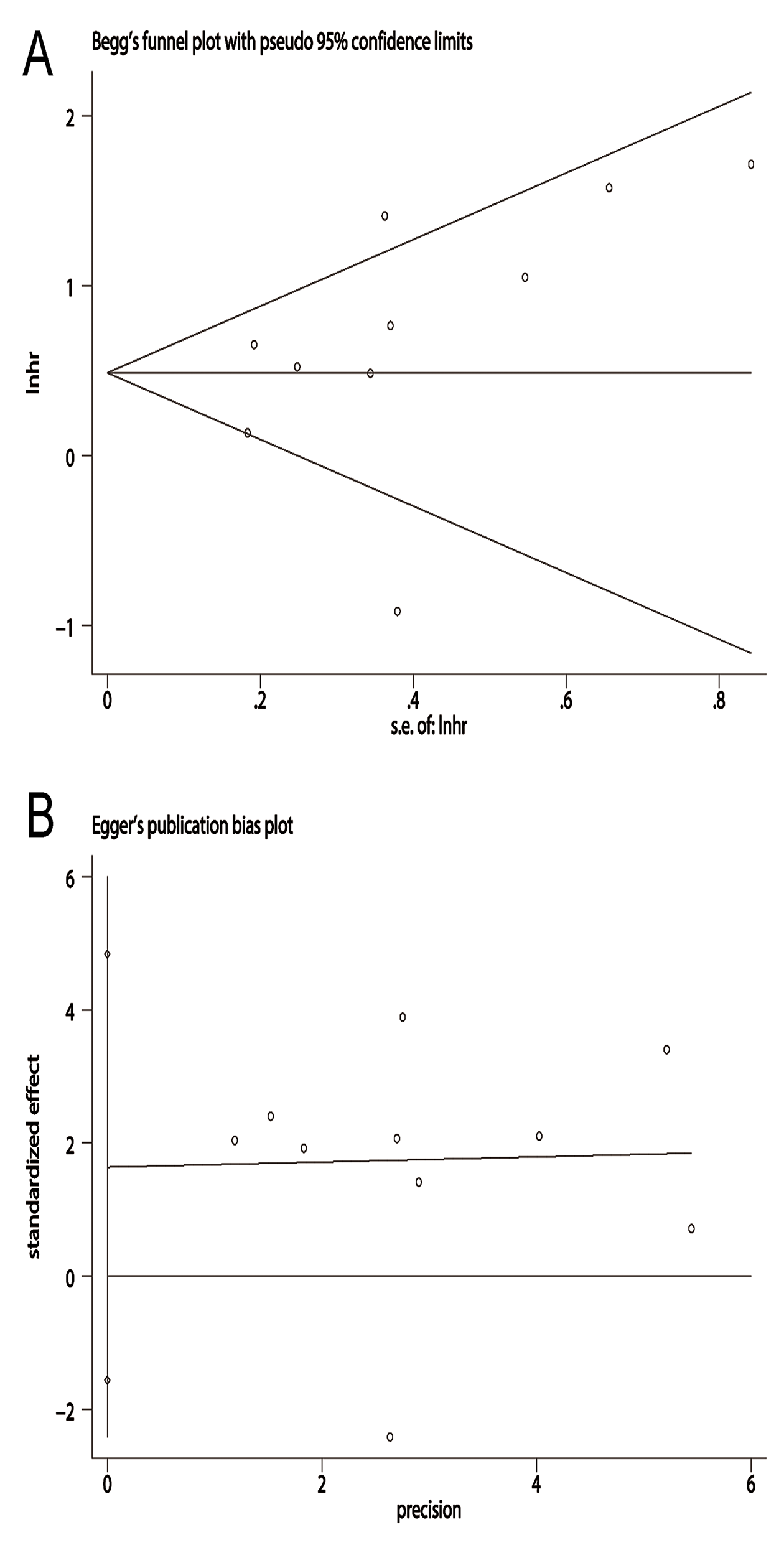

Supplement: Supplementary file 1 — Additional file 1: Figure S1. Funnel plot of publication bias for (A) OS and (B) PFS. [file 12935_2018_609_MOESM1_ESM.tif]

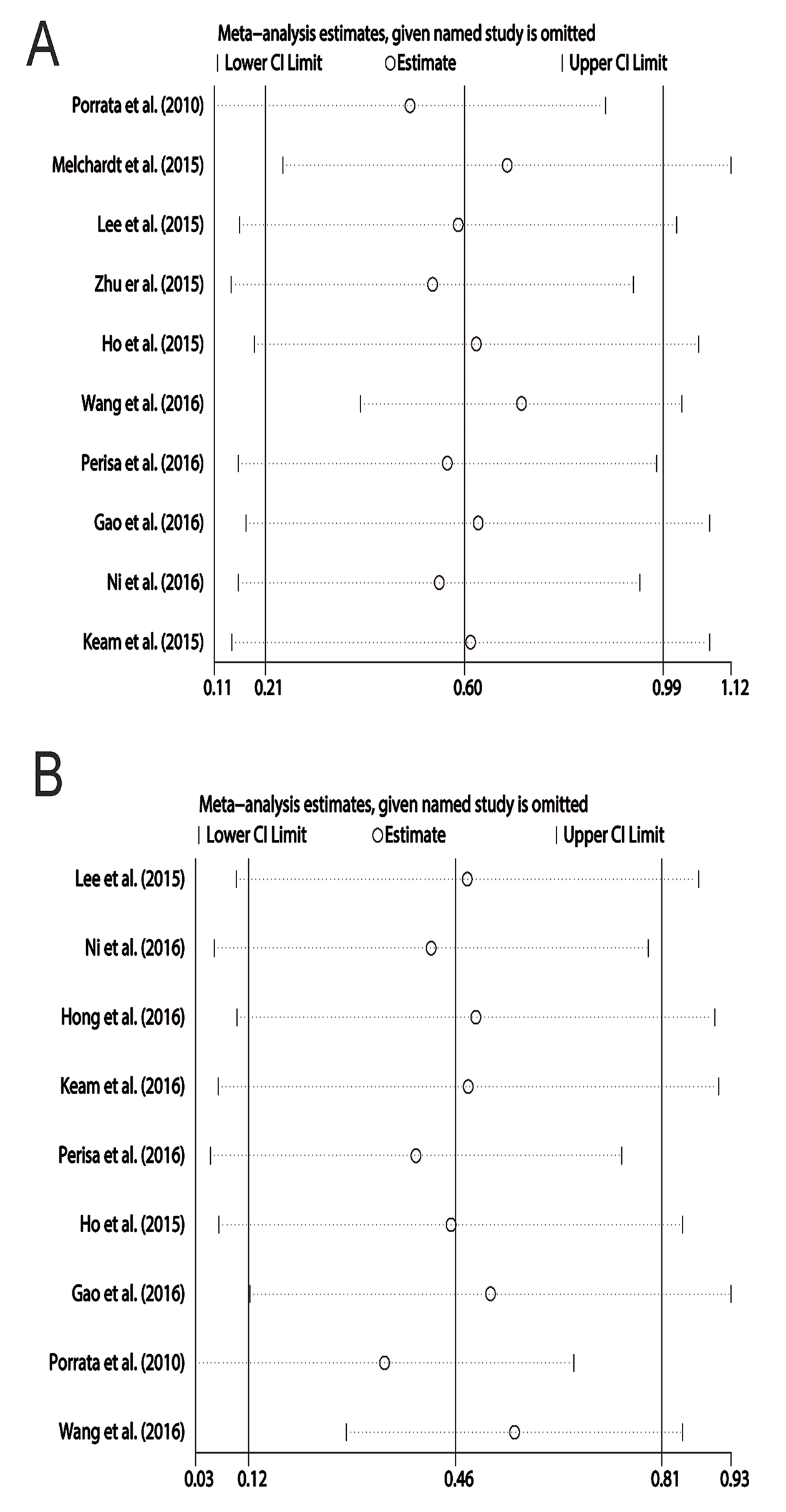

Supplement: Supplementary file 2 — Additional file 2: Figure S2. Sensitivity analysis of studies concerning NLR and (A) OS and (B) PFS. [file 12935_2018_609_MOESM2_ESM.tif]
